# Supplementary material for: Experimental entanglement swapping through single-photon χ(2) nonlinearity
Source: Nat Commun. 2025 Oct 7;16:8720. doi: 10.1038/s41467-025-63785-5 (PMC12504699; doi:10.1038/s41467-025-63785-5)
Supplement: Supplementary file 1 — Supplementary Information [file 41467_2025_63785_MOESM1_ESM.pdf]

Supplementary information for "Experimental entanglement swapping through single-photon $\chi^{(2)}$ nonlinearity"

Yoshiaki Tsujimoto[1*], Kentaro Wakui[1], Tadashi Kishimoto[1], Shigehito Miki[2], Masahiro Yabuno[2], Hirotaka Terai[1,2], Mikio Fujiwara[1], Go Kato[1]

[1]National Institute of Information and Communications (NICT), 4-2-1 Nukui-kita-machi, Koganei, 184-8795, Tokyo, Japan.
[2]National Institute of Information and Communications (NICT), 588-2 Nishi-ku Iwaoka, Kobe, 651-2492, Hyogo, Japan.

*Corresponding author(s). E-mail(s): tsujimoto@nict.go.jp;

SUPPLEMENTARY NOTE 1: MODEL OF SFG-BASED ENTANGLEMENT SWAPPING

We present a detailed procedure for calculating the theoretical visibilities of the swapped state using the realistic model shown in Supplementary Fig. 1. We assume that each EPS consists of two two-mode squeezed vacua, and the initial state is given by $|\psi_{in}\rangle = |\psi\rangle_{ad} \otimes |\psi\rangle_{be}$, where

$$|\psi\rangle_{ad} := \sqrt{1-\gamma_{1H}^2}\sum_{k=0}^{\infty}\frac{1}{k!}(\gamma_{1H}\hat{a}_H^\dagger\hat{d}_H^\dagger)^k|\text{vac}\rangle \otimes \sqrt{1-\gamma_{1V}^2}\sum_{l=0}^{\infty}\frac{1}{l!}(\gamma_{1V}\hat{a}_V^\dagger\hat{d}_V^\dagger)^l|\text{vac}\rangle \quad (1)$$

and

$$|\psi\rangle_{be} := \sqrt{1-\gamma_{2H}^2}\sum_{k=0}^{\infty}\frac{1}{k!}(\gamma_{1H}\hat{b}_H^\dagger\hat{e}_H^\dagger)^k|\text{vac}\rangle \otimes \sqrt{1-\gamma_{2V}^2}\sum_{l=0}^{\infty}\frac{1}{l!}(\gamma_{1V}\hat{b}_V^\dagger\hat{e}_V^\dagger)^l|\text{vac}\rangle. \quad (2)$$

Here, the photon-number distributions are characterized by the average photon numbers as $\gamma_{1H} = \sqrt{\mu_{1H}/(1+\mu_{1H})}$ for example. Hereafter, we consider the events where

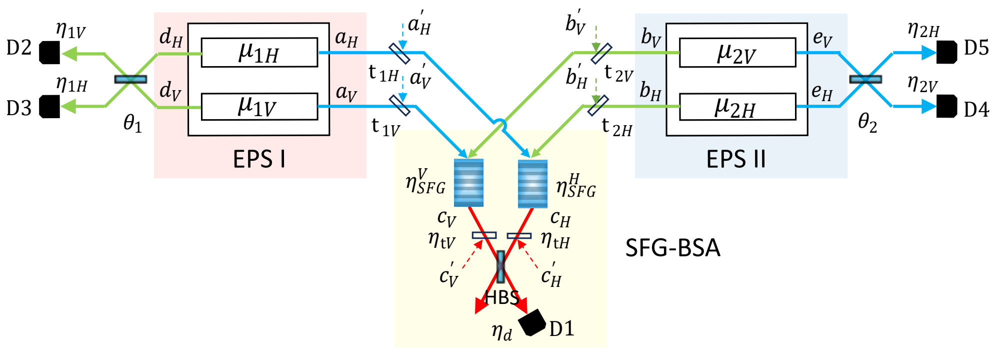

Supplementary Fig. 1 Realistic model of SFG-based entanglement swapping. Each EPS consists of two photon pair sources. The polarization correlation measurements on the swapped state are performed by the beamsplitters in the polarization DOF followed by photon detections using threshold detectors.

up to a total of three photon pairs are produced in $|\psi_{in}\rangle$. Optical losses in mode a_H, a_V, b_H and b_V are simulated by virtual beamsplitters (BSs) whose transmittance are t_{1H}, t_{1V}, t_{2H} and t_{2V}, respectively. For example, the optical loss operation in mode a_H is represented by

$$\mathcal{L}_{a'_H}(|\psi_{in}\rangle\langle\psi_{in}|) = \mathrm{Tr}_{a'_H}[\hat{U}_{a'_H}|\psi_{in}\rangle\langle\psi_{in}| \otimes |\mathrm{vac}\rangle_{a'_H}\langle\mathrm{vac}|_{a'_H}\hat{U}^\dagger_{a'_H}], \tag{3}$$

where a'_H is an ancillary mode and $\hat{U}_{a'_H}$ is the unitary operator of the BS whose transmittance is t_{1H}, which satisfies $\hat{U}_{a'_H}\hat{a}^\dagger_H\hat{U}^\dagger_{a'_H} = \sqrt{t_{1H}}\hat{a}^\dagger_H + \sqrt{1-t_{1H}}\hat{a'}^\dagger_H$ and $\hat{U}_{a'_H}|\mathrm{vac}\rangle = |\mathrm{vac}\rangle$. The quantum state after experiencing the optical losses is given by

$$\hat{\rho}_\mathrm{L} = \mathcal{L}_{a'_H} \circ \mathcal{L}_{a'_V} \circ \mathcal{L}_{b'_H} \circ \mathcal{L}_{b'_V}(|\psi_{in}\rangle\langle\psi_{in}|). \tag{4}$$

Since the threshold detectors are used in the later photon detection, we only need to consider the subspace of $\hat{\rho}_\mathrm{L}$ where the number of photons in $|\psi_{in}\rangle$ and $\langle\psi_{in}|$ are equal. As a next step, the SFG operation is performed on the photons in mode a_H, a_V, b_H and b_V. We note that Eq. (2) in the main text is not enough to express the SFG operation in this model, since it is derived under the condition that the number of input photons is at most two. Thus, we need to derive the SFG operation on the three-photon inputs. Moreover, we consider that the coupling constant χ in Eq. (1) in the main text depends on the polarization of the inputs, which we define $\chi_{H(V)}$ for $H(V)$-polarized input, respectively. Considering only the first order of χ_H and χ_V in $U = e^{-iH'\tau/\hbar}$, the corresponding operation is approximated by

$$U \sim -i\hat{H}'\tau/\hbar = \tau(\chi_H\hat{a}_H\hat{b}_H\hat{c}^\dagger_H + \chi_V\hat{a}_V\hat{b}_V\hat{c}^\dagger_V) - \mathrm{H.c.}. \tag{5}$$

We exemplify the dynamics for the SFG operations involving three photons. As the first case, we consider the case where two photons $\hat{a}^\dagger_i$ and $\hat{b}^\dagger_i$ in an identical polarization

state and a photon $\hat{a}_j^\dagger$ in a polarization orthogonal to them are input, e.g. $\hat{a}_H^\dagger \hat{a}_V^\dagger \hat{b}_H^\dagger |\mathrm{vac}\rangle$. Performing Supplementary Eq. (5), we obtain

$$(-i\hat{H}'\tau/\hbar)\hat{a}_H^\dagger \hat{a}_V^\dagger \hat{b}_H^\dagger |\mathrm{vac}\rangle = \sqrt{\eta_{\mathrm{SFG}}^H}\hat{a}_V^\dagger \hat{c}_H^\dagger |\mathrm{vac}\rangle, \tag{6}$$

where $\eta_{\mathrm{SFG}}^H := (\chi_H \tau)^2$. As the second case, we consider the case where two photons $(\hat{a}_i^\dagger)^2$ in an identical polarization state and photon $\hat{b}_j^\dagger$ in a polarization orthogonal to that of a photon a are input. For example, $(\hat{a}_H^\dagger)^2 \hat{b}_V^\dagger |\mathrm{vac}\rangle$ is transformed into

$$(-i\hat{H}'\tau/\hbar)(\hat{a}_H^\dagger)^2 \hat{b}_V^\dagger |\mathrm{vac}\rangle = 0. \tag{7}$$

As the third case, we consider the case where the three input photons are in an identical polarization state. For example, $\frac{1}{\sqrt{2}}(\hat{a}_H^\dagger)^2 \hat{b}_H^\dagger |\mathrm{vac}\rangle$ is transformed into

$$(-i\hat{H}'\tau/\hbar)\frac{1}{\sqrt{2}}(\hat{a}_H^\dagger)^2 \hat{b}_H^\dagger |\mathrm{vac}\rangle = \sqrt{\frac{\eta_{\mathrm{SFG}}^H}{2}}(1 + \hat{a}_H^\dagger \hat{a}_H)\hat{a}_H^\dagger (1 + \hat{b}_H^\dagger \hat{b}_H)\hat{c}_H^\dagger |\mathrm{vac}\rangle \tag{8}$$

$$= \sqrt{2\eta_{\mathrm{SFG}}^H}\hat{a}_H^\dagger \hat{c}_H^\dagger |\mathrm{vac}\rangle, \tag{9}$$

which shows that the SFG efficiency is doubled compared to the case where $\hat{a}_H^\dagger \hat{a}_V^\dagger \hat{b}_H^\dagger |\mathrm{vac}\rangle$ is the input. The SFG photons in mode c_H and c_V experience optical losses $\mathcal{L}_{c'_H}$ and $\mathcal{L}_{c'_V}$, respectively. The projection of the SFG photon on $|D\rangle$ and $|A\rangle$ is simulated by mixing the photons in mode c_H and c_V by a virtual HBS in polarization degree of freedom followed by photon detections by threshold detectors. Since we consider the case where at most one SFG photon is emitted, POVM elements of the projective measurements on $|D\rangle$ and $|A\rangle$ are approximated by $\Pi_c^D \sim \eta_d |D\rangle_c \langle D|_c$ and $\Pi_c^A \sim \eta_d |A\rangle_c \langle A|_c$, respectively, where η_d is the quantum efficiency of D1. The unnormalized quantum state after detecting the A-polarized SFG photon is given by

$$\hat{\rho}_{\mathrm{SFG}} = \mathrm{Tr}_{a_H, a_V, b_H, b_V, c_H, c_V}[\Pi_c^A \mathcal{L}_{c'_H} \circ \mathcal{L}_{c'_V}((-i\hat{H}'\tau/\hbar)\hat{\rho}_{\mathrm{L}}(-i\hat{H}'\tau/\hbar)^\dagger)]. \tag{10}$$

Finally, polarization correlation measurements on the photons in mode d and e are performed. Similarly to the projective measurement on the SFG photon, the H- and V-polarized components are mixed by virtual BSs in polarization degree of freedom whose transmittance is $\cos^2\theta_1$ and $\cos^2\theta_2$ for the photons in mode d and e, respectively, and are detected by the threshold detectors. We exemplify the POVM element of photon detection at D3 as

$$\hat{\Pi}_{d_H}(\theta_1) = \sum_{n=1}^{2} \frac{1}{n!}(1 - (1 - \eta_{1H})^n)\hat{U}_{\mathrm{BS}}(\theta_1)(\hat{d}_H^\dagger)^n |\mathrm{vac}\rangle\langle \mathrm{vac}|(\hat{d}_H)^n \hat{U}_{\mathrm{BS}}^\dagger(\theta_1). \tag{11}$$

Here, the sum of n is limited to 2, since the maximum number of photons in a single mode is 2 in this model. $\hat{U}_{\mathrm{BS}}(\theta_1)$ is the unitary operator of the BS, which satisfies $\hat{U}_{\mathrm{BS}}(\theta_1)\hat{d}_H^\dagger \hat{U}_{\mathrm{BS}}^\dagger(\theta_1) = \cos\theta_1 \hat{d}_H^\dagger + \sin\theta_1 \hat{d}_V^\dagger$ and $\hat{U}_{\mathrm{BS}}(\theta_1)|\mathrm{vac}\rangle = |\mathrm{vac}\rangle$. When Z(X)-basis

measurement is performed, θ_1 and θ_2 are set to be $0(\pi/4)$, respectively. For example, the coincidence probability between D3 and D5 heralded by D1 is given by

$$P_{HH}^{\mathrm{SFG}}(\theta_1,\theta_2) = \mathrm{Tr}[\hat{\Pi}_{d_H}(\theta_1)\hat{\Pi}_{e_H}(\theta_2)\hat{\rho}_{\mathrm{SFG}}]. \tag{12}$$

Here the subscript "HH" comes from the fact that D3 and D5 detect H-polarized components after the FPBSs. (See Fig. 2 in the main text)

In addition to the influence of the multiphoton creation considered above, there are accidental coincidences caused by the dark counts of the detectors. Since the dark count probabilities of D2, D3, D4, and D5 are sufficiently smaller than their photon detection probabilities, only the effect of the dark count in D1 is considered in this model. The coincidence probability between D3 and D5 heralded by the dark count in D1 is given by $R_d\tau_W(P_{HH}^{\mathrm{Acd}}(\theta_1,\theta_2) - P_{HH}^{\mathrm{SFG}}(\theta_1,\theta_2))$, where $R_d\tau_W$ is the dark count probability of D1, and

$$P_{HH}^{\mathrm{Acd}}(\theta_1,\theta_2) = \mathrm{Tr}[\hat{\Pi}_{d_H}(\theta_1)\hat{\Pi}_{e_H}(\theta_2)\mathrm{Tr}_{aH,aV,bH,bV}[|\psi_{in}\rangle\langle\psi_{in}|]] \tag{13}$$

is the accidental coincidence between D3 and D5. By using Supplementary Eq. (12)

Supplementary Table 1 The experimental parameters necessary for calculating $V_{\mathrm{Z}}^{\mathrm{th}}$ and $V_{\mathrm{X}}^{\mathrm{th}}$.

$*$	μ_{1*}	η_{1*}	t_{1*}	μ_{2*}	η_{2*}	t_{2*}	η_{SFG}^{*}	η_{t*}	η_d
H	0.060	0.097	0.44	0.080	0.070	0.56	2.31×10^{-8}	0.43	0.85
V	0.050	0.11	0.48	0.061	0.10	0.57	2.35×10^{-8}	0.40	0.85

and Supplementary Eq. (13), the visibilities in Z and X basis are respectively given by

$$V_{\mathrm{Z}}^{\mathrm{th}} = \frac{P_{HH}(0,0) + P_{VV}(0,0) - P_{HV}(0,0) - P_{VH}(0,0)}{P_{HH}(0,0) + P_{VV}(0,0) + P_{HV}(0,0) + P_{VH}(0,0)} \tag{14}$$

and

$$V_{\mathrm{X}}^{\mathrm{th}} = \frac{P_{HV}(\frac{\pi}{4},\frac{\pi}{4}) + P_{VH}(\frac{\pi}{4},\frac{\pi}{4}) - P_{HH}(\frac{\pi}{4},\frac{\pi}{4}) - P_{VV}(\frac{\pi}{4},\frac{\pi}{4})}{P_{HV}(\frac{\pi}{4},\frac{\pi}{4}) + P_{VH}(\frac{\pi}{4},\frac{\pi}{4}) + P_{HH}(\frac{\pi}{4},\frac{\pi}{4}) + P_{VV}(\frac{\pi}{4},\frac{\pi}{4})}, \tag{15}$$

where $P_{ij}(\theta_1,\theta_2) := P_{ij}^{\mathrm{SFG}}(\theta_1,\theta_2)(1 - R_d\tau_W) + R_d\tau_W P_{ij}^{\mathrm{Acd}}(\theta_1,\theta_2)$ with $i,j \in \{H,V\}$. Here, $V_{\mathrm{Z}}^{\mathrm{th}}$ and $V_{\mathrm{X}}^{\mathrm{th}}$ are functions of the experimental parameters summarized in Supplementary Table 1. Substituting the experimental parameters, we obtain $V_{\mathrm{Z}}^{\mathrm{th}} = 0.78$ and $V_{\mathrm{X}}^{\mathrm{th}} = 0.76$, respectively.

SUPPLEMENTARY NOTE 2: COMPARISON OF SFG-BSA AND LINEAR OPTICAL BSA

In this section, we quantitatively show the superiority of the SFG-BSA over the linear optical BSA (LO-BSA). We introduce the model of the LO-BSA as shown in Supplementary Fig. 2. We assume perfect HOM interference and no dark count at the BSA.

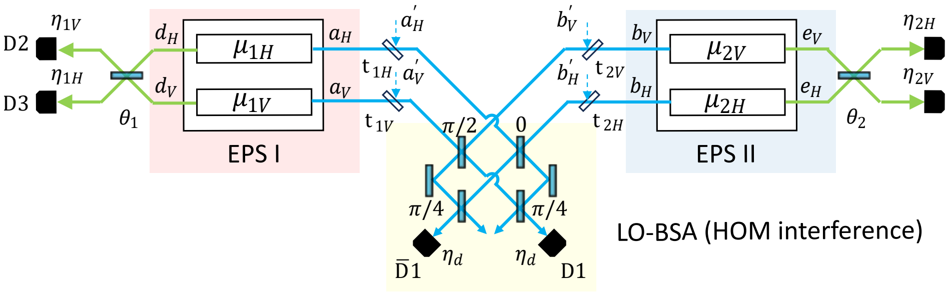

Supplementary Fig. 2 Model of linear optical entanglement swapping. Linear optical BSA is realized by mixing the photons in mode a and b with a polarization beamsplitter and then performing projective measurements in X basis on each output.

The unnormalized quantum state just before the BSA is given by $\hat{\rho}_{\text{L}}$ in Supplementary Eq. (4). Here, we assume the symmetric transmittance as $t_{1H} = t_{1V} = t_{2H} = t_{2V} = t$ for simplicity. The photons in mode a and b are then mixed by a PBS, which is simulated by mixing the H- and V-polarized components of the photons using two beamsplitters $\hat{U}^{H}_{\text{BS}}(0)$ and $\hat{U}^{V}_{\text{BS}}(\pi/2)$, respectively as

$$\hat{\rho}_{\text{PBS}} = (\hat{U}^{H}_{\text{BS}}(0) \otimes \hat{U}^{V}_{\text{BS}}(\pi/2))\hat{\rho}_{L}(\hat{U}^{H}_{\text{BS}}(0) \otimes \hat{U}^{V}_{\text{BS}}(\pi/2))^{\dagger}, \tag{16}$$

where $\hat{U}^{H}_{\text{BS}}(0)$ and $\hat{U}^{V}_{\text{BS}}(\pi/2)$ satisfy $\hat{U}^{H}_{\text{BS}}(0)\hat{a}^{\dagger}_{H}(\hat{U}^{H}_{\text{BS}}(0))^{\dagger} = \hat{a}^{\dagger}_{H}$, $\hat{U}^{H}_{\text{BS}}(0)\hat{b}^{\dagger}_{H}(\hat{U}^{H}_{\text{BS}}(0))^{\dagger} = \hat{b}^{\dagger}_{H}$, $\hat{U}^{V}_{\text{BS}}(\pi/2)\hat{a}^{\dagger}_{V}(\hat{U}^{V}_{\text{BS}}(\pi/2))^{\dagger} = \hat{b}^{\dagger}_{V}$, $\hat{U}^{V}_{\text{BS}}(\pi/2)\hat{b}^{\dagger}_{V}(\hat{U}^{V}_{\text{BS}}(\pi/2))^{\dagger} = \hat{a}^{\dagger}_{V}$ and $\hat{U}^{H}_{\text{BS}}(0)|\text{vac}\rangle = \hat{U}^{V}_{\text{BS}}(\pi/2)|\text{vac}\rangle = |\text{vac}\rangle$. Finally, the four-fold coincidences among D1, $\bar{\text{D}}1$, (D2 or D3) and (D4 or D5) are measured. Here, A- and D-polarized components are respectively detected by D1 and $\bar{\text{D}}1$ whose quantum efficiencies are η_d. For example, the four-fold coincidence probability among D1, $\bar{\text{D}}1$, D3 and D5 is given by using the POVM elements defined in Supplementary Eq. (11) as

$$P^{\text{LO}}_{HH}(\theta_1, \theta_2) = \text{Tr}[\hat{\Pi}_{dH}(\theta_1)\hat{\Pi}_{eH}(\theta_2)\hat{\Pi}_{b_H}(\pi/4)\hat{\Pi}_{a_V}(\pi/4)\hat{\rho}_{\text{PBS}}]. \tag{17}$$

The visibilities of the swapped state are calculated by replacing $P_{ij}(\theta_1, \theta_2)$ in Eqs. (14) and (15) with $P^{\text{LO}}_{ij}(\theta_1, \theta_2)$.

In the following, we assume that the average photon number of each two-mode squeezed vacuum is the same as $\mu_{1H} = \mu_{1V} = \mu_{2H} = \mu_{2V} = 0.05$ and the photon detection efficiencies at the LO-BSA are unity as $\eta_d = 1$ for simplicity. The visibilities V^{th}_{Z} and V^{th}_{X} versus optical loss $(1 - t)$ with $\eta_{1H} = \eta_{1V} = \eta_{2H} = \eta_{2V} = 1$ and $\eta_{1H} = \eta_{1V} = \eta_{2H} = \eta_{2V} = 0.1$ are plotted in Supplementary Fig. 3**a** and **b**, respectively. The visibilities decrease as the photon losses increase. This is because the portion of fake success events caused by two photons from one EPS to enter the LO-BSA and be a coincidence between D1 and $\bar{\text{D}}1$ increases as the photon losses increase.

The visibilities obtained by the SFG-BSA *without* dark counts are calculated by replacing $P_{ij}(\theta_1, \theta_2)$ in Eqs. (14) and (15) with $P^{\text{SFG}}_{ij}(\theta_1, \theta_2)$. Here, we assume $\mu_{1H} = \mu_{1V} = \mu_{2H} = \mu_{2V} = 0.05$, $t_{1H} = t_{1V} = t_{2H} = t_{2V} = t$ and $\eta_{TH} = \eta_{TV} = \eta_d = 1$

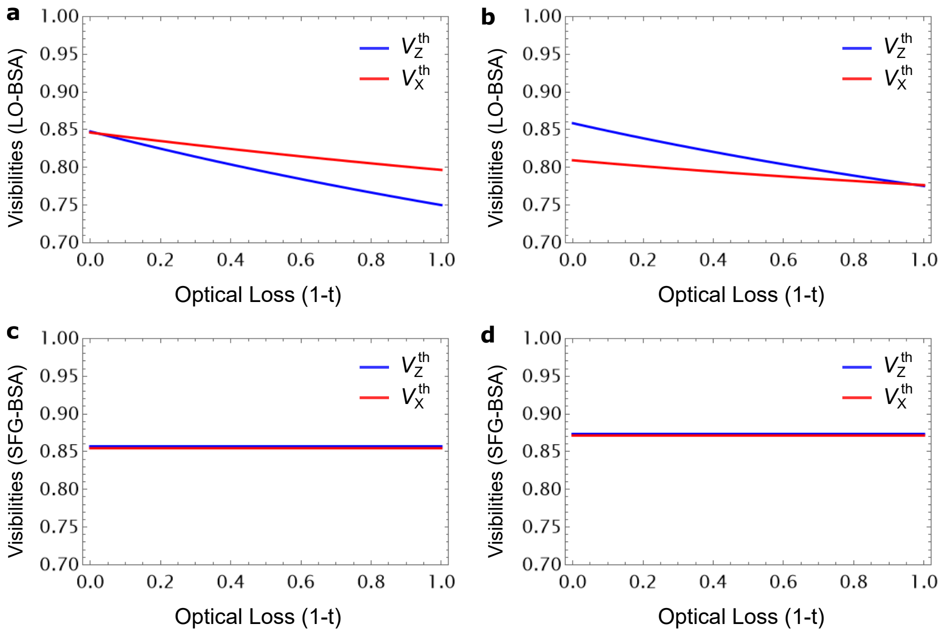

Supplementary Fig. 3 Visibilities versus optical loss. a, b Entanglement swapping using LO-BSA with **a** $\eta_{1H} = \eta_{1V} = \eta_{2H} = \eta_{2V} = 1$ and **b** $\eta_{1H} = \eta_{1V} = \eta_{2H} = \eta_{2V}$=0.1. **c,d** Entanglement swapping using SFG-BSA with **c** $\eta_{1H} = \eta_{1V} = \eta_{2H} = \eta_{2V} = 1$ and **d** $\eta_{1H} = \eta_{1V} = \eta_{2H} = \eta_{2V} = 0.1$.

as in the case of the LO-BSA. The visibilities V_Z^{th} and V_X^{th} versus optical loss $(1-t)$ with $\eta_{1H} = \eta_{1V} = \eta_{2H} = \eta_{2V} = 1$ and $\eta_{1H} = \eta_{1V} = \eta_{2H} = \eta_{2V} = 0.1$ are plotted in Supplementary Fig. 3**b** and **d**, respectively. We see that the visibilities are insensitive to the optical losses. This is because the fake success events related to the optical losses, which occur in the LO-BSA, are rejected by the SFG-BSA. As a result, the visibilities remain high despite the effects of optical losses.

SUPPLEMENTARY NOTE 3: EXPERIMENTAL RESULTS OF SFG-BASED QUANTUM TELEPORTATION

We show all density matrices and raw counts obtained in the quantum teleportation experiment using SFG-BSA. The density matrices of the input states are shown in Supplementary Fig. 4. The density matrices and raw counts of the teleported states are shown in Supplementary Fig. 5 **a-c** and **d-f**, respectively.

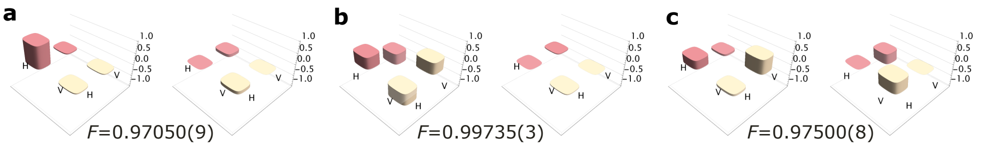

Supplementary Fig. 4 Input polarization states. **a**, **b**, **c** Density matrices of the H-, A-, and R-polarized states, respectively.

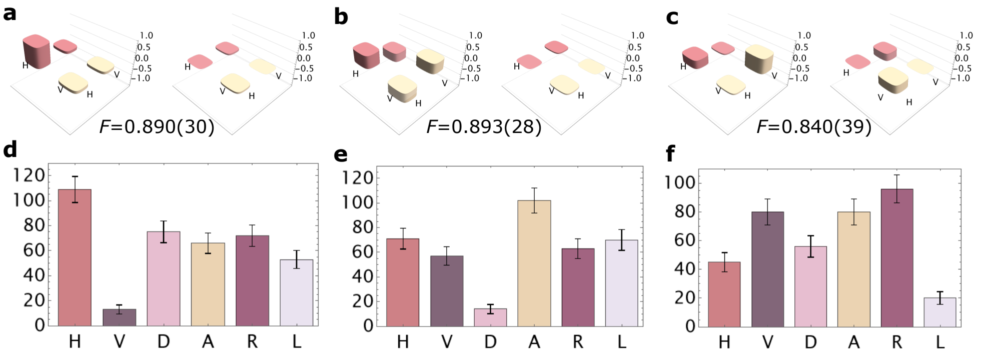

Supplementary Fig. 5 Teleported states and raw detection counts. **a**, **b**, **c** Density matrices of the teleported states for the H-, A-, and R-polarized input states, respectively. **d**, **e**, **f** Raw detection counts of the teleported photons for the H-, A-, and R-polarized input states, respectively. The error bars were calculated assuming the Poisson statistics.

SUPPLEMENTARY NOTE 4: SFG-BASED QUANTUM TELEPORTATION WITH LARGE-PHOTON-NUMBER INPUTS

We used coherent light with an average photon number of 7.5×10^4 to prepare the input states and perform the quantum teleportation experiment. We show the density matrices and raw counts of the output states in Supplementary Fig. 6. The fidelities of the output states for H-, A- and R-polarized inputs are 0.973(2), 0.954(2) and 0.917(3), respectively. Interestingly, unlike the case of the linear optical BSA, we observe the "quantum-teleportation-like" operation even if a number of multiple photons are contained in the input coherent light as reported in Ref. [1]. This result can be interpreted as follows. Assuming that the average intensities of the H- and V-polarized portion of the input coherent light in mode b are respectively given by $|\alpha|^2$ and $|\beta|^2$, where the polarization is expressed as $(\alpha\boldsymbol{H}+\beta\boldsymbol{V})/\sqrt{|\alpha|^2+|\beta|^2}$, and are sufficiently large, $\hat{b}_{H(V)}$ in Eq. (1) in the main text is replaced with $\alpha(\beta)\in\mathbb{C}$, respectively, as

$$\hat{H}_{\rm QFC} = i\hbar\chi(\alpha\hat{a}_H\hat{c}_H^\dagger + \beta\hat{a}_V\hat{c}_V^\dagger) + \text{H.c.}, \tag{18}$$

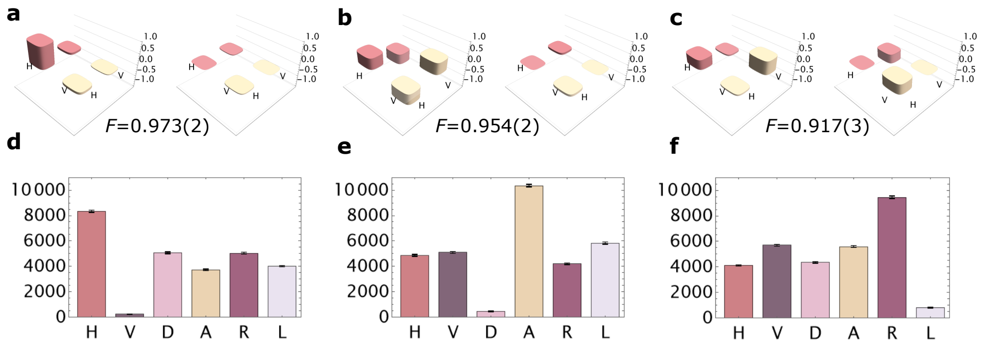

Supplementary Fig. 6 Output states and raw detection counts. **a**, **b**, **c** Density matrices of the output states after the QFC for the H-, A-, and R-polarized input states, respectively. **d**, **e**, **f** Raw detection counts of the output photons after the QFC for the H-, A-, and R-polarized input states, respectively. The error bars were calculated assuming the Poisson statistics.

which corresponds to the Hamiltonian of the quantum frequency conversion (QFC) [2–4], between mode a and c. Here, the input light in mode b is regarded as pump light. The unitary transformation $\hat{U}_{\mathrm{QFC}} = e^{-i\hat{H}_{\mathrm{QFC}}\tau/\hbar}$ of $\hat{a}_{H(V)}$ is represented by [5]

$$\hat{U}_{\mathrm{QFC}}\hat{a}_{H(V)}\hat{U}^{\dagger}_{\mathrm{QFC}} = \cos\left(|\alpha(\beta)|\chi\tau\right)\hat{a}_{H(V)} + e^{i\arg\alpha(\beta)}\sin\left(|\alpha(\beta)|\chi\tau\right)\hat{c}_{H(V)}. \tag{19}$$

In the weak pump regime where $|\alpha(\beta)|\chi\tau \ll 1$, the right hand side of Supplementary Eq. (19) is approximated by $\hat{a}_{H(V)} + \sqrt{\eta_{\mathrm{SFG}}}\alpha(\beta)\hat{c}_{H(V)}$. Thus, after the QFC followed by the detection of a D-polarized SFG photon in mode c, we obtain ${}_c\langle D|\hat{U}_{\mathrm{QFC}}|\Phi^+\rangle_{ad} \propto \alpha|H\rangle_d + \beta|V\rangle_d$, which shows that the polarization information of the input coherent light is transferred to the photon in mode d. We emphasize that the above dynamics is different from the quantum teleportation. Therefore, in the strong pump regime, the conversion efficiency is no longer proportional to the pump intensity, and the situation is rather similar to the polarization insensitive QFC [5–7] where no polarization information of the input (pump) light remains in the output photon.

SUPPLEMENTARY NOTE 5: FABRICATION OF PPLN/W3

A ridge waveguide was formed by mechanical processing using a 3-inch z-cut MgO-doped PPLN substrate with a period of 18.2 μm. The PPLN substrate was bonded to an undoped $LiNbO_3$ substrate with an adhesive layer as an under-cladding. The PPLN substrate was thinned to a thickness of 7.2 μm by a lapping and polishing process. The variation in PPLN thickness along the waveguide was within approximately 0.5 μm. In the next step, the ridge structure was formed using a dicing saw. The ridge width was 8 μm. To minimize chipping and waveguide width variation, the finest diamond blade was used to cut the PPLN film at low cutting speed, resulting in low propagation

loss. The waveguide was cut to an interaction length of 6.3 cm. The end faces of the waveguide device were anti-reflective coated for both telecom and 780 nm light.

SUPPLEMENTARY NOTE 6: SPATIAL PROFILES AND SPECTRAL DISTRIBUTIONS

The spatial profiles and spectral distributions of the photons that are involved in the single-photon SFG process are shown in Supplementary Fig. 7**a** and **b**, respectively. The spatial profiles of the photons in mode a and b were simulated by injecting laser light at 1535 nm and 1585 nm into PPLN/W1 and 2, respectively. The spatial profile of the photon in mode c was simulated by measuring the spatial profile of the SFG light at 780 nm generated by PPLN/W3. The spectral distributions of the photons in mode a and b were estimated by measuring the diffraction spectrum of the VHG and transmission spectrum of Filter I by sweeping the center wavelength of the tunable laser, respectively. The spectral distribution of the photon in mode c was estimated by measuring the phase matching bandwidth of PPLN/W3. The FWHMs of the bandwidths of the photons in mode a and c were estimated to be 55 GHz and 39 GHz, respectively. As shown in Supplementary Fig. 7**b**, the bandwidth of the photon in mode b was much broader than those of the photons in mode a and c. In fact, a narrow band pass filter was not necessary for the photon in mode b in our setup because the components that were outside the phase matching bandwidth of PPLN/W3 and bandwidth of the photon in mode a do not contribute to the SFG process.

SUPPLEMENTARY NOTE 7: APPLICATION TO LOOPHOLE-FREE BELL TEST

We describe here the details of the simulation used to evaluate the performance of SFG-BSA for a loophole-free Bell test and device-independent quantum key distribution (DIQKD). We begin by outlining the procedure for performing the Bell test using the quantum state heralded by SFG-BSA. As illustrated in Supplementary Fig. 1, under the condition that D1 clicks, Alice and Bob independently choose their measurement angles $\{\theta_{A1}, \theta_{A2}\} \in \theta_1$ and $\{\theta_{B1}, \theta_{B2}\} \in \theta_2$, respectively, and perform measurements. For each setting, they record all combinations of detection ("click") and non-detection ("no-click") events without any postselection. Each party then applies a locally defined strategy to assign binary outcomes (± 1) to each detection event. Since each party has four possible local outcomes—(i) only D3 (D5) clicks, (ii) only D2 (D4) clicks, (iii) both detectors click, and (iv) neither detector clicks—there are $2^4 = 16$ possible strategies per party. In our simulation, we adopt the following representative strategy: outcome (i) is assigned -1, while outcomes (ii), (iii), and (iv) are assigned $+1$. For example, when Alice and Bob choose measurement settings θ_{A1} and θ_{B1}, respectively, the probability that both obtain the outcome -1 which we denote $P(-1,-1|\theta_{A1},\theta_{B1})$ is obtained by the probability that detectors D3 and D5 click, while detectors D2 and D4 do not. Similarly, the conditional probabilities $P(+1,-1|\theta_{A1},\theta_{B1})$, $P(-1,+1|\theta_{A1},\theta_{B1})$, and $P(+1,+1|\theta_{A1},\theta_{B1})$ are also computed from the corresponding detection events. The Clauser–Horne–Shimony–Holt (CHSH)

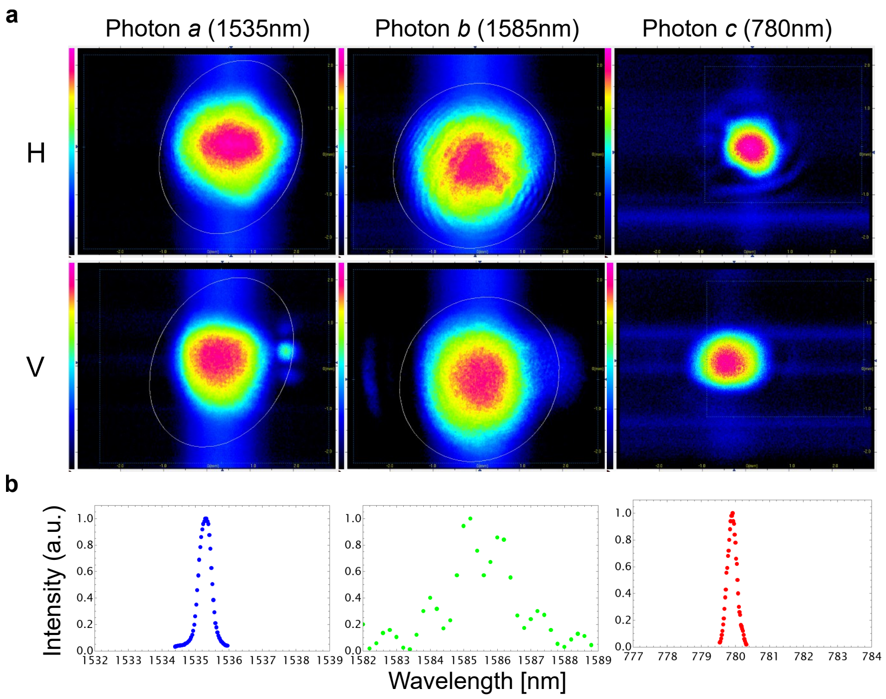

Supplementary Fig. 7 Spatial profiles and spectral distributions. Spatial profiles (**a**) and spectral distributions (**b**) of the photons in mode a, b and c.

value S is defined by

$$S = \langle A_1B_1\rangle + \langle A_2B_1\rangle + \langle A_1B_2\rangle - \langle A_2B_2\rangle, \tag{20}$$

where $\langle A_iB_j\rangle = P(+1,+1|\theta_{Ai},\theta_{Bj}) + P(-1,-1|\theta_{Ai},\theta_{Bj}) - P(+1,-1|\theta_{Ai},\theta_{Bj}) - P(-1,+1|\theta_{Ai},\theta_{Bj})$. Although the maximal value of $|S|$ is in the upper bound of 2 in the framework of a local realism theory, it can exceed 2 in quantum mechanics. When Alice and Bob perform DIQKD, Alice chooses another measurement angle θ_{A0}, and the raw key is generated under the condition that Alice and Bob choose θ_{A0} and θ_{B1}, respectively. The lower bound of the asymptotic key rate r is represented by [8, 9]

$$r \geq r_{\mathrm{DW}} = 1 - h(Q) - \chi(S), \tag{21}$$

where r_{DW} is the Devetak-Winter rate [10], Q is qubit error rate which is defined by

$$Q = P(+1,-1|\theta_{A0},\theta_{B1}) + P(-1,+1|\theta_{A0},\theta_{B1}), \tag{22}$$

and

$$\chi(S) = h\left[\frac{1+\sqrt{(S/2)^2-1}}{2}\right]. \tag{23}$$

Here, $h(\cdot)$ is the binary entropy defined by $h(x) = -x\log_2 x - (1-x)\log_2(1-x)$.

Next, we calculate the maximum CHSH value S achieved by SFG-BSA. We consider the case where each detector has no dark count and has unity detection efficiency, and the quantum state used for the Bell test is given by $\hat{\rho}_{\mathrm{SFG}}/\mathrm{Tr}[\hat{\rho}_{\mathrm{SFG}}]$ in Supplementary Eq. (10). By optimizing the average photon number of each SPDC source and the measurement angles, we obtain the maximum CHSH value of $S = 2.82843 \sim 2\sqrt{2}$, which is the maximum value allowed by quantum mechanics known as the Tsirelson bound [11] and much higher than the maximum CHSH value achieved by the LO-BSA [12]. By maximizing the CHSH value with decreasing η_{1H}, η_{1V}, η_{2H} and η_{2V} in Supplementary Fig. 1, the minimum detection efficiency allowed for each of Alice's and Bob's detectors is estimated. It is found that $S > 2$ holds for $\eta_{1H} = \eta_{1V} = \eta_{2H} = \eta_{2V} = 0.68$, which is much smaller than 0.911 which is the minimum detection efficiency allowed by LO-BSA [12]. These results show the qualitative difference between SFG-BSA and LO-BSA.

Finally, we estimate the extent to which the SFG efficiency must be improved such that the heralded quantum state exhibits correlations sufficient to violate the Bell–CHSH inequality ($S > 2$) and to yield a positive key rate in DIQKD. In this simulation, we use the experimental parameters in Supplementary Table 1 and the dark count probability of D1 as $R_d\tau_W = 6.7\times10^{-11}$. In our current system, the dark count probability is still much higher than the heralding probability ($\mathrm{Tr}[\hat{\rho}_{\mathrm{SFG}}] = 7.6\times10^{-12}$), which implies that most of the heralding signal is caused by the dark counts in D1. In this situation, the normalized quantum state heralded by the click signal from D1 is given by

$$\hat{\rho}_{\mathrm{herald}} = \frac{\hat{\rho}_{\mathrm{SFG}} + R_d\tau_W(\mathrm{Tr}_{aH,aV,bH,bV}[|\psi_{in}\rangle\langle\psi_{in}|])}{\mathrm{Tr}[\hat{\rho}_{\mathrm{SFG}} + R_d\tau_W|\psi_{in}\rangle\langle\psi_{in}|]}, \tag{24}$$

where $\mathrm{Tr}_{aH,aV,bH,bV}[|\psi_{in}\rangle\langle\psi_{in}|]$ is the uncorrelated quantum state heralded by a dark count. We substituted the experimental parameters in Supplementary Table 1, $R_d = 0.15$ and $\tau_W = 448\times10^{-12}$ for Supplementary Eq. (24), and optimized the measurement angles. Assuming $\eta_{1H} = \eta_{1V} = \eta_{2H} = \eta_{2V} = 1$, we obtain $S = 1.88 < 2$, which indicates that the quantum state heralded by our SFG-BSA would not violate the Bell-CHSH inequality. Nevertheless, it is revealed that $S > 2$ would be achieved if the SFG efficiency is tripled. To apply for DIQKD, further improvement of the SFG efficiency and optimization of the average photon numbers are necessary, because not only $\chi(S)$ in Supplementary Eq. (21) but also $h(Q)$ must be much smaller than 1. In the absence of loss in the SFG-BSA, i.e. $\eta_t\eta_d = 1$, the key rate becomes positive if the SFG efficiency increases by a factor of 50. When the loss in the SFG-BSA is taken into account, an improvement of approximately 140 times in SFG efficiency is required. These levels of improvement are considered realistic in light of recent progress in nonlinear resonators reporting efficiency improvements of two to three orders of magnitude compared to PPLN/W [13, 14].

SUPPLEMENTARY REFERENCES

[1] Kim, Y.-H., Kulik, S.P., Shih, Y.: Quantum teleportation of a polarization state with a complete bell state measurement. Phys. Rev. Lett. **86**, 1370–1373 (2001) https://doi.org/10.1103/PhysRevLett.86.1370

[2] Kumar, P.: Quantum frequency conversion. Opt. Lett. **15**(24), 1476–1478 (1990) https://doi.org/10.1364/OL.15.001476

[3] Tanzilli, S., Tittel, W., Halder, M., Alibart, O., Baldi, P., Gisin, N., Zbinden, H.: A photonic quantum information interface. Nature **437**(7055), 116–120 (2005) https://doi.org/10.1038/nature04009

[4] Ikuta, R., Kusaka, Y., Kitano, T., Kato, H., Yamamoto, T., Koashi, M., Imoto, N.: Wide-band quantum interface for visible-to-telecommunication wavelength conversion. Nature Communications **2**(1), 537 (2011) https://doi.org/10.1038/ncomms1544

[5] Ikuta, R., Kobayashi, T., Kawakami, T., Miki, S., Yabuno, M., Yamashita, T., Terai, H., Koashi, M., Mukai, T., Yamamoto, T., Imoto, N.: Polarization insensitive frequency conversion for an atom-photon entanglement distribution via a telecom network. Nature Communications **9**(1), 1997 (2018) https://doi.org/10.1038/s41467-018-04338-x

[6] Bock, M., Eich, P., Kucera, S., Kreis, M., Lenhard, A., Becher, C., Eschner, J.: High-fidelity entanglement between a trapped ion and a telecom photon via quantum frequency conversion. Nature Communications **9**(1), 1998 (2018) https://doi.org/10.1038/s41467-018-04341-2

[7] Leent, T., Bock, M., Garthoff, R., Redeker, K., Zhang, W., Bauer, T., Rosenfeld, W., Becher, C., Weinfurter, H.: Long-distance distribution of atom-photon entanglement at telecom wavelength. Phys. Rev. Lett. **124**, 010510 (2020) https://doi.org/10.1103/PhysRevLett.124.010510

[8] Acín, A., Brunner, N., Gisin, N., Massar, S., Pironio, S., Scarani, V.: Device-independent security of quantum cryptography against collective attacks. Phys. Rev. Lett. **98**, 230501 (2007) https://doi.org/10.1103/PhysRevLett.98.230501

[9] Pironio, S., Acin, A., Brunner, N., Gisin, N., Massar, S., Scarani, V.: Device-independent quantum key distribution secure against collective attacks. New Journal of Physics **11**(4), 045021 (2009)

[10] Devetak, I., Winter, A.: Distillation of secret key and entanglement from quantum states. Proc. R. Soc. London, Ser. A **461**(2053), 207–235 (2005). The Royal Society

[11] Cirel'son, B.S.: Quantum generalizations of bell's inequality. Letters in Mathematical Physics **4**(2), 93–100 (1980)

[12] Tsujimoto, Y., You, C., Wakui, K., Fujiwara, M., Hayasaka, K., Miki, S., Terai, H., Sasaki, M., Dowling, J.P., Takeoka, M.: Heralded amplification of nonlocality via entanglement swapping. New Journal of Physics **22**(2), 023008 (2020) https://doi.org/10.1088/1367-2630/ab61da

[13] Lu, J., Li, M., Zou, C.-L., Sayem, A.A., Tang, H.X.: Toward 1% single-photon anharmonicity with periodically poled lithium niobate microring resonators. Optica **7**(12), 1654–1659 (2020) https://doi.org/10.1364/OPTICA.403931

[14] Akin, J., Zhao, Y., Misra, Y., Haque, A.K.M.N., Fang, K.: InGaP $\chi(2)$ integrated photonics platform for broadband, ultra-efficient nonlinear conversion and entangled photon generation. Light: Science & Applications **13**(1), 290 (2024)
